# Tensile Deformation and Transverse Strain Behavior of Carbon Black-UHMWPE Composites

**DOI:** 10.3390/ma18112542

**Published:** 2025-05-28

**Authors:** Peder C. Solberg, Douglas W. Van Citters

**Affiliations:** Thayer School of Engineering, Dartmouth College, Hanover, NH 03755, USA

**Keywords:** ultra-high molecular weight polyethylene, composites, plastic deformation, necking, strain

## Abstract

Electrically conductive composites of ultra-high molecular weight polyethylene (UHMWPE) may be of interest as strain sensors for event detection in high-strain scenarios, with potential applications in ballistics or orthopedics. In this study, geometric deformations of electrically conductive composites of UHMWPE were quantified for large plastic strains via physical measurements. These measurements were compared to neat (non-composite) control materials, and to geometrical behaviors predicted under volume conservation assumptions. This study found that material geometry remained close to that predicted by volume conservation at low-to-moderate plastic strains, with differences exceeding 5% only above 100% nominal strain. Materials with higher filler loading experienced a greater increase in measured volume than neat controls, particularly at higher strains. The results suggest that this difference could be due in part to volumetric opening in the composite materials with high filler loading. Finally, necking behavior was observed and quantified in this study, presenting another effect that should be taken into account for future work characterizing the electrical behavior of these materials under large plastic deformations. The results of this study thus lay the foundation for further characterization of these electrically-conductive composites, and to determine their intrinsic electrical properties as a function of strain in particular.

## 1. Introduction

Polymer composites are used for a wide variety of applications, as they have tunable properties and can have advantages including high toughness, chemical resistance, and biocompatibility. Electrically conductive ultra-high molecular weight polyethylene (UHMWPE) composites’ high ductility and ability to integrate well with existing UHMWPE materials (see [1,2]) make them compelling for use as event-detection sensors in certain plastic deformation scenarios. For example, such materials may be useful for adverse event detection in spinal implants or penetration detection in bulletproof UHMWPE vests. Furthermore, the advent of smart fabrics and wearable technology could provide additional opportunities for tough, flexible, electrically conductive materials such as the carbon black-UHMWPE composites covered in this study.

UHMWPE is unique as a base material for composite strain sensors due to its extremely high melt viscosity. This leads to a segregated microstructure, wherein the filler particles exist within distinct filler-rich domains around the outside of the polymer granules. This unique structure allows less conductive filler to be used to achieve the same level of conductivity, as compared to a randomly-dispersed filler [3,4]. For this reason, these materials are of interest for various unloaded electrical applications, such as anti-static materials, current collectors in batteries, fuel cell bipolar plates, supercapacitor plates, and electromagnetic interference (EMI) shielding [5,6,7,8]. Conductive UHMWPE composites may also be useful for loaded or strained applications in fields that already utilize neat UHMWPE for its unique properties, e.g., good energy-absorption and biocompatibility [2,9,10,11]. Regarding the former, UHMWPE is often used in lightweight bullet-proof vests due to its excellent ability to absorb energy from a high-velocity projectile [12,13,14]. Regarding the latter, UHMWPE is used in a variety of applications in the body requiring high toughness, wear resistance, and chemical inertness. For example, UHMWPE has been the bearing material of choice for 60 years in joint replacements [15,16,17]. Furthermore, braided UHMWPE suture is often used in scenarios requiring non-resorbable fixation and good mechanical properties [18]. High-contrast versions of this suture have recently been made available, typically containing carbon black as a colorant [19,20]. This additive, which also can be used to confer electrical conductivity to UHMWPE, has been declared safe for use as a colorant in this in vivo application, at concentrations up to 1 wt.% [21].

With this in mind, electrically conductive composites of UHMWPE could enable new possibilities in these application areas already relying on neat (non-composite) UHMWPE. For example, in the ballistics market, integrating strain sensing could enable rapid emergency response following detection of projectile impact characteristics (e.g., location on body, extent of vest damage, number of impacts, etc.) [22,23]. While some smart armor systems detect stimuli via chemical or acoustic sensors, other smart armor paradigms involve response to mechanical and electronic stimuli [22,24,25]. This is the paradigm most relevant for the materials of interest in this study: a mechanical deformation event causes an electrical response that is detected (via a change in material electrical properties with strain) and transmitted to a communication system. Carbon-polymer composites are particularly of interest for this type of application, as stark changes in sensor resistance can occur with strain due to disconnections in the conductive network or formation of small cracks or voids with the material [26]. In orthopedics, detection of large strain events via a similar type of system may aid in rapid identification and response to failures in patients with spinal implants. For example, some recent treatment paradigms for proximal junctional kyphosis (PJK) involve the use of polymeric tethers to stabilize the spine and prevent over-straining of spinal segments adjacent to implants [27]. Incorporating strain sensing elements into the polymeric tethers used for PJK augmentation could allow failures or impending failure events to be detected and treated much sooner.

These application areas motivate a better understanding of UHMWPE composites’ electrical behavior in plastic deformation scenarios. Specifically, to successfully use these materials in event detection scenarios, changes to their extrinsic and intrinsic electrical properties with strain must be understood. While understanding extrinsic properties, like resistance and gauge factor, is very important for assessing viability as a sensing material [28], quantifying these behaviors involves a relatively straightforward process. On the other hand, determining changes to the material’s intrinsic resistivity as a function of strain requires the geometric parameters of the sensing domain to be known at every value of strain. Specifically, the sample thickness and width must be known to find the cross-sectional area of the sample through which current will flow in electrical applications. For these reasons, this study will not focus on characterization of electrical properties in these materials with strain, but rather will lay the groundwork necessary for such a study by quantifying the geometric deformation behavior of these materials in the plastic regime.

It is known that longitudinal strains cause the greatest deformation in the longitudinal direction [29], but deformations in the transverse direction are also important, as they cause changes in the cross-sectional area of the sample. In the elastic regime, for UHMWPE, the ratio of transverse strain to axial strain (Poisson’s ratio) is approximately 0.46 [1]. In the plastic regime, molecular stretching effects are overtaken by permanent changes via crystalline deformation and molecular alignment in the amorphous and crystalline domains [30]. Typically, the ratio of transverse strain to axial strain in the plastic regime increases to around 0.5 in many materials, indicative of volume-conserving deformation effects taking over [31,32,33]. However, in composites of UHMWPE, the integration of nanoparticles can cause a greater propensity for the formation of micro-voids in the materials. The degree of interfacial bonding of filler-rich domains and the bulk polymeric domains affects the ability for stress transfer to occur in the materials [29], and can thus lead to regions of volumetric opening in these polymer composites. Overall increases in sample volume due to this effect will inevitably change the transverse deformations and the overall cross-sectional area of the materials at high strain. These deformation effects must be understood before determining the behavior of electrons traveling through the changing cross-sections of these materials at high strains.

Thus, to better understand the ability of these materials to be integrated into event-detection sensing platforms, this study seeks to elucidate several specific behaviors of interest in carbon black-UHMWPE composites. In the first section, we will address three related questions: (1) How closely can these candidate sensors’ transverse deformations be predicted based on volume conservation principles in the plastic regime? This will provide an understanding of the extent to which longitudinal strain can be used to predict changes in transverse geometries. Longitudinal strain can be measured without requiring strain measurement apparatuses in the sensing domain, which may disrupt the electrical behavior of the material. Thus, assuming volume conservation can allow for more facile characterization of intrinsic electrical property evolution with strain. If this approach is to be used, the extent of its validity must be understood across a range of strain. The second question relates to the effect of various material treatments: (2) What is the influence of filler concentration on the geometric parameters of these composite materials under large plastic deformations? In these segregated network composites, voids may form in the carbon black-rich domains between polymer granules, especially with increases in strain. This effect is hypothesized to be greater at higher filler concentrations and higher levels of strain, and can be quantified by finding increases in the volume of the material as a function of strain and filler loading. Lastly, due to the potential for necking behavior in the plastic regime of these highly ductile composites, it is important to understand local changes in the deformation behavior of long gauge regions. Such behavior could ultimately affect the electrical behavior of these materials under strain. While this study does not seek to define that effect on electrical behavior, it does seek to answer this third question: (3) When does the onset of material necking occur as a sample is strained, and how does this necking progress under further increases in the plastic deformation?

Answering these three questions may not only give the ability to define intrinsic electrical properties as a function of strain, but can also elucidate some of the microstructural behaviors responsible for the macroscopic behavior of these phase-segregated composites. To that end, microscopic imaging of the materials will be included to allow further interpretation of the results. The resulting understanding of structure–property relationships in these phase-segregated composites may also be relevant for application areas utilizing non-conductive composites of UHMWPE in high-deformation scenarios. Thus, this study has broad relevance in the area of high-viscosity, phase-segregated polymer composites. With that said, this study will not seek to rigorously interpret the results in the context of plasticity theory, but instead will focus on rigorously characterizing the material to the extent necessary for practical implementation of electrical resistivity measurements in the sample types of interest.

## 2. Materials and Methods

### 2.1. Material Manufacturing Process

UHMWPE powder (Celanese GUR 1020, Celanese Corp, Irving, TX, USA) and pigment carbon black (PCB) powder (Asbury 5388 Pigment Black, Asbury, NJ, USA) were weighed for several desired filler concentrations and placed in a glass mixing vessel. Powders were acoustically mixed at 100 G acceleration for 3 min at 60 Hz in a Resodyn LabRAM I resonant acoustic mixer to achieve dispersive mixing of the powders (Resodyn, Butte, MT, USA). This was followed by lower-velocity mixing wherein the cylindrical mixing vessel was mounted to a motorized plate and tumbled end-over-end for 2 min at 40 RPM to achieve a higher level of distributive mixing of the two powder phases. Two 140 g batches were used to make each billet of material, as the volume of the mixing vessels was limited. This mixing procedure was performed on neat control samples as well to ensure consistency with the composites.

After mixing, powders were compression molded in the horizontal section of a die described by Reinitz et al. [34] at 14 MPa and 175 °C for 2.5 h. This time and temperature are similar to those used for consolidation in Favreau et al. and Reinitz et al. [34,35,36], and this consolidation pressure is within the 8–20 MPa range common for processing of UHMWPE composites [2,37,38,39]. Heating took 20–30 min, during which time the horizontal die plunger was actuated occasionally to push out air pockets and decrease powder volume until a fully dense mass was obtained and held at temperature for the remaining ~2 h. Consolidated billets with approximate dimensions of 15 × 5 × 5 cm were removed immediately via an ejection plunger and allowed to cool overnight at room temperature before being processed further. 

Billets were cut with a bandsaw to obtain rough-cut sections of appropriate size to make tensile test samples approaching plane stress conditions. A heavy-duty sledge microtome (Leica/Reichert-Jung Polycut S, Leica Microsystems, Wetzlar, Germany) was used to cut tensile samples to a thickness of 0.200 mm. From these thin sections, a custom razor blade jig was used to cut a rectangular sample approximately 3.3 mm in width and 50 mm in length. The long axis of the samples was oriented in the direction of compression from the compression molding process. These samples, which have a large difference between thickness and width, were chosen as the through-thickness stresses are very low, while the through-width stresses are slightly higher. Differences in geometric deformation across these two dimensions could reveal underlying effects of transverse stress on geometric behavior. Rectangular samples were chosen as they have a consistent initial cross-sectional area between grips and can allow for electrical properties to be determined throughout that rectangular region using embedded electrodes in the Instron grips. The purpose of this study is, in effect, to understand and quantify the limitations associated with this approach.

### 2.2. Material Testing Process

**Figure 1:**
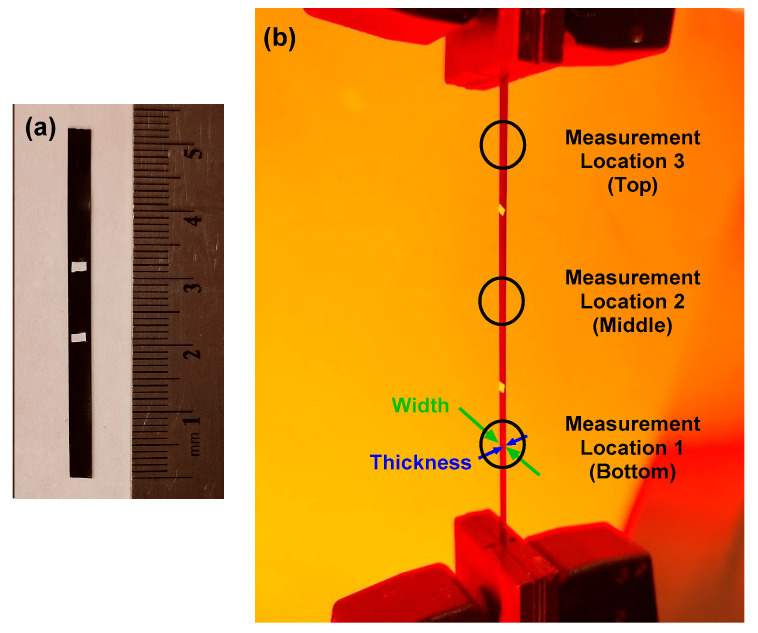
(**a**) Undeformed sample, showing scale. (**b**) Measurement types (sample width, sample thickness) and approximate locations. Width measurements were made with calipers and thickness measurements were made with a micrometer. One measurement was taken at each of the three locations, for each of three samples for each material treatment. This image is of a strained sample, partway through the test procedure described.

Once prepared, samples were clamped in the grips of an Instron 68SC-2 mechanical load frame (Instron, Norwood, MA, USA) with the distance between grips set to 30 mm, edge to edge. Pneumatic clamping pressure was set at 5 bar to minimize sample slippage during straining. A custom Instron test method was developed to increase nominal strain in the samples by 3 mm at each of 15 setpoints, such that the final setpoint occurred with the sample at 90 mm, or 300% of its original length. Setpoints included the following nominal strains: 0, 10, 20, 30, 40, 50, 60, 70, 80, 90, 100, 150, 200, 250, 300%. Prior to testing, small white tape marks were affixed to the sample approximately 12 mm apart to demarcate consistent locations for measurements to be taken during the tests. The sample was then clamped in the Instron with these marks centered between the grips. Following removal of any slack in the sample, initial dimensions were measured. For all thickness measurements, a Mitutoyo micrometer was used with a resolution of 0.001 mm (Mitutoyo America Corp., Aurora, IL, USA, C/N 293-340-30). Width measurements required use of calipers to gently contact opposing edges of the sample to get an accurate reading without bending the sample. For this, Tesa Digi-Cal calipers were used with a resolution of 0.01 mm (Brown & Sharpe TESA, Renens, Switzerland). At each strain setpoint, three measurements of thickness and three measurements of width were taken, in the bottom third, middle third, and top third of the sample, as shown in Figure 1. The material regions immediately next to the grips were avoided, as material geometry differed slightly there from the main gauge region of the sample. Crosshead speed was set to 25.4 mm/min, with samples held at each strain setpoint for 80 s to allow ample time for careful measurements to be taken. Four composite material treatments were investigated, with carbon black filler concentrations of 2.5 wt.%, 5 wt.%, 7.5 wt.%, and 10 wt.%. A neat (0 wt.%) version of the material was used as a control, as it does not contain the filler-rich domains which could lead to significant intergranular void formation at higher strains and concentrations in the composites. For each material treatment, three samples were tested.

### 2.3. Prediction of Material Deformation Using Conservation of Volume

Volume conservation in the plastic deformation regime is a common assumption [31,32,33], which can be expressed as the sum of the incremental plastic strains in three orthogonal directions [40]:

(1) $${d\epsilon }_{P,11}+{d\epsilon }_{P,22}+{d\epsilon }_{P,33}=0.$$

In terms of true strain expressions, this can be written in terms of a rectangular prism’s geometric parameters length, thickness, and width (*L*, *t*, and *w*, respectively):

(2) $$\ln\left(\frac{L}{L_{0}}\right)+\ln\left(\frac{t}{t_{0}}\right)+\ln\left(\frac{w}{w_{0}}\right)=0.$$

Assuming that the two transverse strains (for thickness and width) are equivalent (which is consistent with volume conservation), one gets:

(3) $$-\ln\left(\frac{L}{L_{0}}\right)=2\ln\left(\frac{t}{t_{0}}\right).$$

Then, taking the exponential of each side, one finds:

(4a) $$\frac{1}{e^{\ln\left(\frac{L}{L_{0}}\right)}}=e^{2\ln\left(\frac{t}{t_{0}}\right)}=e^{\ln{\left(\frac{t}{t_{0}}\right)}^{2}}.$$

Simplifying, one finds:

(4b) $$\sqrt{\frac{1}{\left(\frac{L}{L_{0}}\right)}}=\frac{t}{t_{0}},$$

and finally, for thickness,

(5) $$t=\frac{t_{0}}{\sqrt{\frac{L}{L_{0}}}}.$$

By the same logic, for width,

(6) $$w=\frac{w_{0}}{\sqrt{\frac{L}{L_{0}}}}.$$

Equations (5) and (6) take the initial, known sample thickness (*t*_0_), width (*w*_0_), and length (*L*_0_), and use the engineering strain values to calculate the thickness and width at every value of strain. The theoretical validity of this equation only holds for the plastic regime, where volume conservation (incompressibility—effectively, a Poisson’s ratio, or lateral stretch ratio, of 0.5 [41,42]) generally tends to hold [31,32,33], with some slight deviations known to occur due to formation of voids [41]. In the elastic regime, the Poisson’s ratio for UHMWPE is known to be closer to 0.46 [1]. Thus, it is known that the behavior of UHMWPE materials will not adhere to volume conservation in the elastic regime. To understand how well the rectangular samples of interest behaved according to volume conservation principles at large plastic strains, this study involved comparison of experimental geometries to those predicted under volume conservation assumptions.

For example, the experimental thickness *t_E_* at some experimental strain was compared to the thickness predicted via volume conservation (found with Equation (5)):

(7) $$D_{t_{EvsP}}=\frac{t_{E}-t_{P}}{\left(\frac{t_{E}+t_{P}}{2}\right)}\times 100$$

where $D_{t_{EvsP}}$ is the percent difference in the experimental thickness as compared to the thickness predicted with volume conservation. A similar comparison was made for width values. To provide comparisons of sample volumes, the following equation was used to compute volume:

(8) V=L×t×w,

where *L* is the distance between grips (i.e., sample length), thickness *t* and width *w* are defined at any value of strain of interest.

### 2.4. Calculating Effect of Volumetric Opening in the Material

To find the increase in volume of the composites attributable to volumetric changes in the material, the difference between the volume of the composite at the strain of interest and the neat control at that same strain was found. The initial difference in volume between the composite and the neat control was subtracted out, as samples’ initial geometry could vary slightly. The resulting value was normalized to the initial volume of the composite and multiplied by 100 to provide a percentage increase in volume of the composite of interest as compared to the neat control. Thus, the following equation was used to perform this calculation:

(9) $$\left(\left(V_{C,\varepsilon }-V_{N,\varepsilon }\right)-\left(V_{C,i}-V_{N,i}\right)\right)/\left(V_{C,i}\right)\times 100=I_{C\;vs\;N}\;$$

where subscript *C* represents the composite, *N* is the neat control, ε represents “strained geometry”, i represents “initial geometry”, and $I_{CvsN}$ represents the percent increase in this measure of volume for the composite material of interest over of the neat control. Note the result is unitless, as it should be for a percentage. In a very similar manner, to compare the increase in sample volume of the various strained materials to those predicted by volume conservation, the value of $V_{N,\varepsilon }$ in the above equation was replaced with $V_{N,i}$, as the volume of the neat comparator material does not deviate from its initial volume under volume conservation assumptions.

### 2.5. Other Calculations

Other analyses included in this study involved performing simple computations on the data, such as normalizing to an initial geometrical parameter of the sample, such as thickness *t*,

(10) $$t_{normalized}=\frac{t_{strained}}{t_{initial}},$$

A similar computation was completed to find normalized width. To directly compare transverse geometrical changes in thickness and width of the composite materials versus the neat control, the difference was found in the normalized values. For example, for thickness,

(11) $$d_{t_{norm_{C\;vs\;N}}},\;=t_{norm,C}-t_{norm,N}$$

where $d_{t_{norm_{C\;vs\;N}}}$ is the difference in the normalized thickness for the composite material versus the neat control. As a note, since this value is the difference between two values normalized to initial thickness, if the result were multiplied by 100, it would represent a percent difference relative to the original normalized geometries (because *t_norm,initial_* = 1). Another computation performed in this study involved finding the total amount of the original sample length experiencing some amount of deformation during the test:

(12) $${L_{deformed}=L}_{undeformed}-L_{original}.$$

The next section briefly describes the method used to quantify the geometrical heterogeneity across the three measurement locations, indicating necking.

### 2.6. Quantifying Necking Behavior

To quantify the geometrical heterogeneity across the three measurement locations, the normalized cross-sectional area was found at each of those three measurement locations, for all strains:

(13) $$A_{norm}=\frac{A_{strained}}{A_{initial}}$$

Lastly, to quantify the extent of geometric heterogeneity due to necking, the three measurement locations were characterized to determine the thinnest region (greatest necking), the thickest region (least necking), and the third (moderate) region, as determined by the average normalized cross-sectional area over all strains. Then, the following equation was applied to the thickest and thinnest of the three measurement locations:

(14) $${D_{A}}_{thick\;vs\;thin}={(A}_{normthickest}-A_{normthinnest})\times 100$$

where ${D_{A}}_{thick\;vs\;thin}$ is the percent difference in normalized cross-sectional area between the thickest region and the thinnest region. Plotting this measure over all strains gave an indication of the extent of necking as a function of sample elongation.

### 2.7. Scanning Electron Microscopy

Scanning electron microscopy (SEM) was performed to observe relevant microstructural phenomena in the composite materials. To accomplish this, a Thermo Fisher Helios 5CX DualBeam microscope (Thermo Fisher Scientific, Waltham, MA, USA) was used following sputter coating of samples with 6 nm of gold/palladium coating. Accelerating voltage ranged from 2 kV to 5 kV, and working distance ranged from 4 to 15 mm. To capture images of strained samples, they were first clamped in place at strains of interest (~20% and ~100% nominal strain) on a custom aluminum/brass jig permitting in situ imaging of strained samples, then gold/palladium coated, and imaged while still clamped in place on the jig.

### 2.8. Statistical Analysis

To ascertain whether carbon black concentration had a significant effect on the calculated differences between experimental and predicted (volume-conserving) geometries at a high strain, ordinary one-way ANOVA analyses were performed for the data at 300% nominal strain. Separate ANOVA tests were completed on thickness and width data. The mean values of thickness and width, respectively, were used for each sample (the mean was found using the three measurement locations). Those three sample values were used in the ANOVA to represent the sample-to-sample variation in the value of percent difference between the experimental value and the predicted value. A *p*-value less than 0.05 was considered statistically significant. This analysis was completed in GraphPad Prism version 10.2.2 for Windows (GraphPad Software, Boston, MA USA).

## 3. Results and Discussion

### 3.1. Transverse Dimensional Changes as a Function of Strain

**Figure 2:**
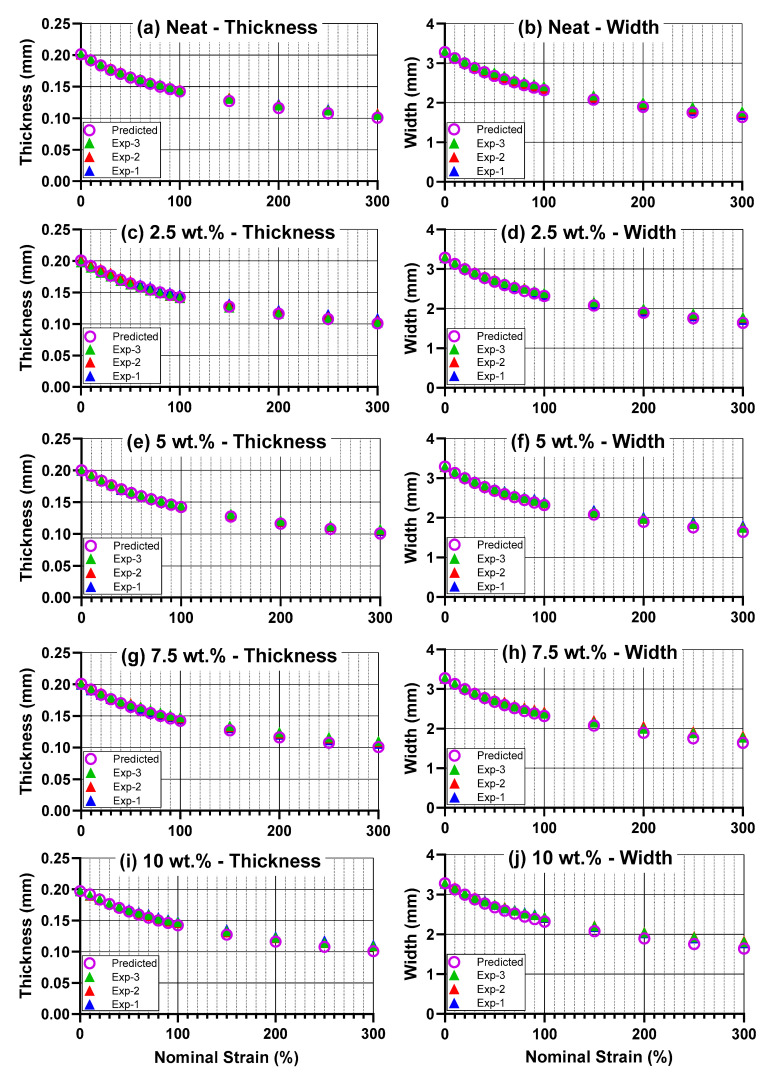
(**a**–**j**) Transverse geometrical properties as a function of strain, including the three experimental measurements (“Exp-1”, “Exp-2”, “Exp-3”) and the geometry predicted using volume conservation principles based on the initial geometry and the longitudinal strain (“Predicted”). Left column—thickness as predicted across all concentrations of interest, Right column—width as predicted across all concentrations.

Figure 2 displays the transverse geometric results as a function of nominal strain, with differences between experimental data and predicted data (volume-conserving) displayed in Table 1. Overall, these results show that volume conservation principles can predict material geometry in these materials within 2% up to at least 30% nominal strain, and within 5% up to 100% nominal strain. Materials with higher filler loading, e.g., 10 wt.%, experienced an increase in deviation from the predicted line, particularly at higher strains. To investigate this in a statistical sense, one-way ANOVA tests were performed on the data from 300% strain to determine if this deviation from the predicted value of thickness or width was significantly different across the carbon black concentrations tested. With a resulting *p* = 0.0087 for the thickness ANOVA and *p* = 0.046 for the width ANOVA, concentration was shown to have a significant effect on transverse geometry, as manifested in the comparison of experimental values to those predicted by volume conservation. Furthermore, the measurements used to find the data in Table 1 exhibited a high degree of accuracy and precision: sample-to-sample standard deviations remained under 1.5% of the measured dimension for thickness and under 2.2% for width across all values of strain. This indicates a high level of precision in these measurements. With regard to accuracy, the similar results across two different measurement modalities (thickness, measured with a micrometer, and width, measured with calipers) suggest that the measurements are accurately capturing the physical changes in sample dimensions.

The fact that the experimental thickness and widths are generally always higher than those predicted via conservation of volume assumptions is expected for several reasons. For example, elastic strain is still present in the materials in the plastic regime, but is not expected to change significantly beyond the yield strain (which occurs before the first 10% nominal strain setpoint [1]), and would be expected to cause a slight upward shift in the data across all strains tested here. However, the differences between experimental and predicted data increase at higher values of strain and, therefore, must be attributed to something else.

One potential factor related to the experimental setup is sample deformation from the grips. While rectangular samples provide the benefit of a consistent initial cross-sectional area over a traditional dog bone-shaped tensile sample, they do allow for a small amount of sample deformation to occur within the grips—that is, outside of the defined 30 mm gauge length. When this inevitably occurs with increases in strain, some additional volume of material will be added to the gauge region from the gripped region. This will present as an increase in sample thickness and width over that which would be predicted using volume conservation principles. While this effect will not be consistent with strain, it is expected to be consistent across the different material treatments studied here, as they have similar bulk properties. For all material treatments in this study, this effect was quantified by measuring the length of undeformed material remaining on each end of the sample following testing and subtracting it from the original sample length. This gave the total amount of the original sample length experiencing some amount of deformation during the test. The results indicated a difference of less than 0.5% between the averages of this metric for the neat, 2.5 wt.%, 5 wt.%, and 7.5 wt.% samples, while the deformed region in the 10 wt.% sample had 2% less length than the neat. This suggests consistency of this effect across most material treatments, with a slight deviation at high concentration. At 10 wt.%, having a smaller total length of deformed material suggests that less material is entering the gauge region from the grips, perhaps due to a slight difference in elastic modulus in that material. This would lessen the effect of volume being added to the sample from the grips region for this material treatment. With this in mind, there are further differences to be accounted for between the composite material and the neat control, especially at high strain. The next section will explore this further.

### 3.2. Volumetric Changes as a Function of Strain and Filler Concentration

**Figure 3:**
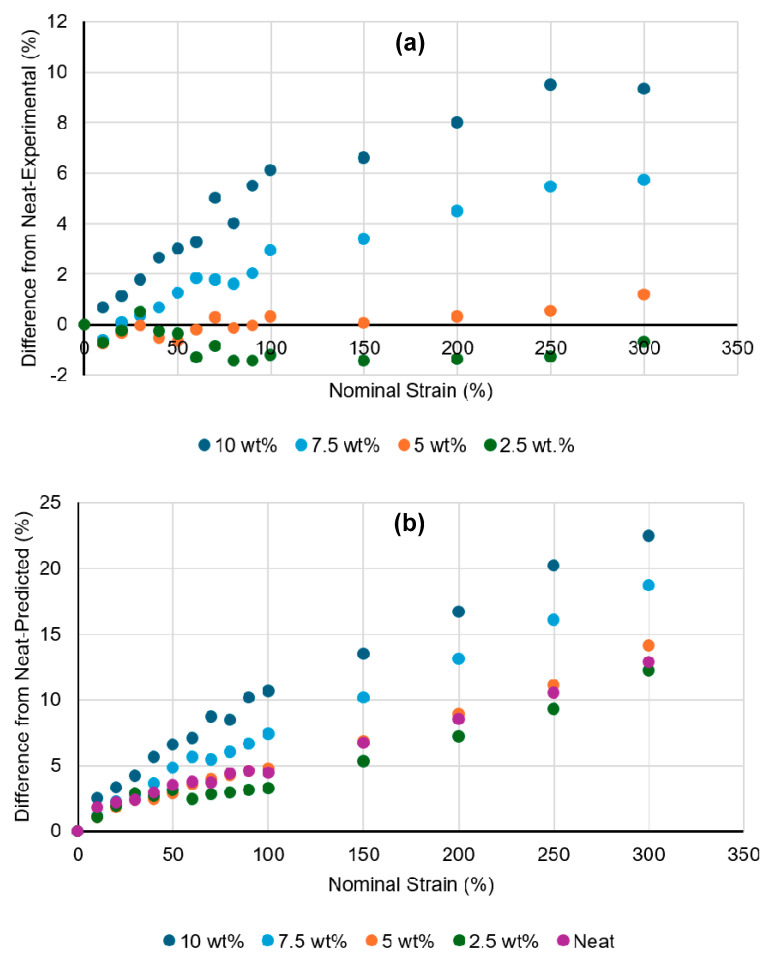
Percent difference in sample volume between grips for each composite material treatment as compared to (**a**) the actual neat values, and (**b**) the data predicted via volume conserving principles based on initial neat geometry. All calculations were performed using the averaged values from the three samples for each material treatment.

**Figure 4:**
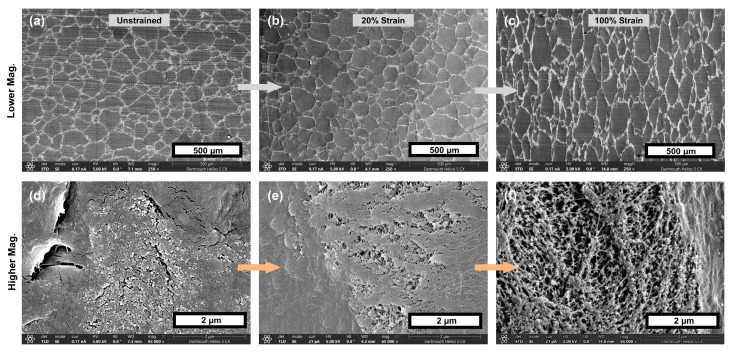
(**a**–**f**) SEM images demonstrating volumetric openings increasing with strain in the 10 wt.% material. As seen in the bottom row, the amount of void space increases with increasing strain, with 0% nominal strain in (**d**), 20% nominal strain in (**e**), and 100% nominal strain in (**f**).

It is hypothesized that, with higher strain and higher levels of carbon black additive, intergranular void space will form to a greater extent in the composites and lead to higher material volume. With this in mind, the volume of each sample calculated from the length, width, and thickness at each strain setpoint was found, and the percent difference between this volume and the neat control material’s volume at equivalent strain was found (after subtracting out any initial difference in volume from the neat material—see Equation (7) in the Methods (Section 2.3)). Since the effect discussed in the previous paragraph (volume being “added” to the gauge region from the grips region) was similar for the composites as compared to the neat material, the difference calculated here already accounts for that effect. Thus, the results in Figure 3a should solely represent the difference in actual material volume when comparing each composite with the neat control at equivalent strain. These results demonstrate that, as hypothesized, the extent of volumetric opening in the materials tends to increase with higher concentrations and higher strains in these materials. Figure 3b shows a similar comparison, but using the difference between each material treatment as compared to the neat sample geometry as predicted with volume conservation. While an error propagation analysis was deemed unnecessary to highlight the general trends of this data, reproducibility of these results can be understood in a general sense by referring to the sample-to-sample standard deviations of the two measured values used to compute the Figure 3 data (thickness and width), as provided in the Supplemental Materials Table S1. SEM images are included in Figure 4 demonstrating this volumetric opening effect at the 10 wt.% concentration. The 10 wt.% material was selected for visualization as it had the most significant volumetric opening in the strained condition. These regions of volumetric opening, which are more like micro-voids than like cracks, are distributed around the outside of the granule boundaries. They generally have greater openings observed at the top and bottom of the polymer granules, as those are regions where the plane of opening is normal to the direction of strain, and thus most readily causes Mode I opening [43]. In the highest concentration, the size of these openings in the vertical direction was at least on the order of 1 micron at 100% strain; additional volumetric opening is likely present but may be obscured by the surrounding material Buklovskyi et al. demonstrated that the granule boundaries of carbon-UHMWPE composites increase in size with increasing carbon black filler concentration [44], so this behavior in which larger filler concentration leads to higher volumetric opening is to be expected.

**Figure 5:**
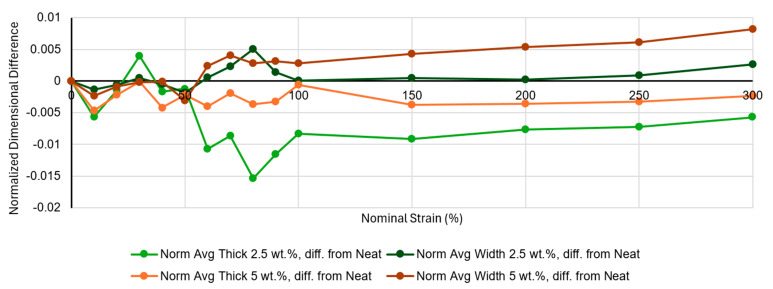
Difference of composite normalized thickness and width, compared to neat controls, for 2.5 wt.% (green/dark green) and 5 wt.% (orange/brown). Normalizations were performed by dividing average thickness at each strain by average initial thickness for each material treatment, then doing the same for width.

**Figure 6:**
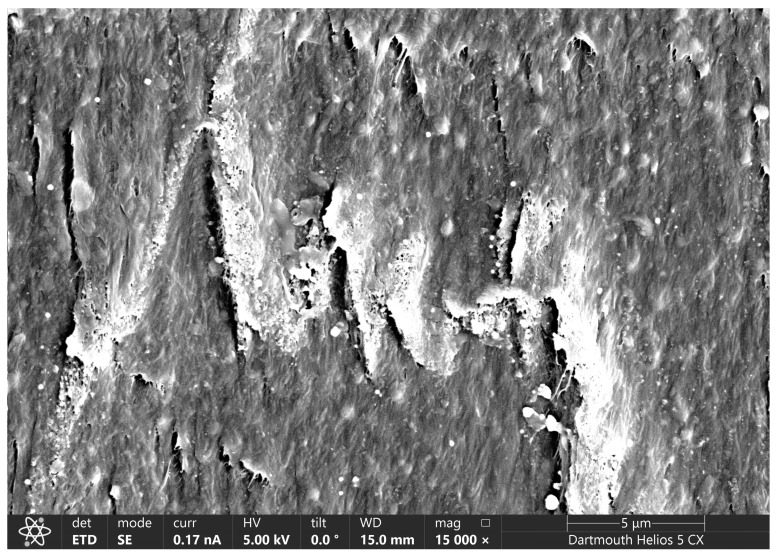
SEM image of a 2.5 wt.% sample, strained to approximately 100% nominal strain, demonstrating the slight topography that can form at filler-rich granule boundary regions.

Curiously, as seen in Figure 3a, the 2.5 wt.% material progresses to have a smaller volume than the neat control material over moderate strains. While the magnitude of this difference is small and might simply suggest that the 2.5 wt.% material behaves similarly to neat, the effect appears consistent, and also is present to a certain degree in the 5 wt.% results. To investigate this behavior, the relative contributions of thickness and width towards the calculated volumes were considered. Figure 5 shows the normalized average thicknesses and widths for neat and 2.5 wt.% materials. The normalized thickness for 2.5 wt.% drops with moderate strain relative to the neat control, while the normalized width does not, suggesting that the lower measured volume of that material compared to the neat control must be due to the greater dimensional decrease in its through-thickness direction. There are several reasons why this could occur because of the different structure exhibited in the composite materials compared to the neat materials. One explanation is that the through-thickness stress, while already low in these thin sections of material, may be able to drop further in the composite materials due to strain relief occurring in the filler-rich granule boundary regions. This could lead to lower thicknesses in the composites than the neat control material. Another observation is that the filler-rich granule boundary regions of the composites appear to have some regions of elevated topography on the surface of the sample (see Figure 6); for a low level of filler concentration, these elevated regions are very narrow, and they may be easily deformed by the micrometer during initial measurements on the sample. This would lead to a slight drop in normalized thickness measurements as compared to the neat control for some of the measurements at lower strain. Thus, the fact that the 2.5 wt.% results in Figure 5 tend to drop below zero at nominal strains below 100% might make sense.

### 3.3. Necking Behavior

The previous sections have investigated the transverse geometrical properties of these materials, in terms of their overall averages. However, it is also important to understand any heterogeneity occurring in the sample deformation, as this could affect interpretation of the materials’ electrical characteristics. Specifically, a region in the sample that deforms to have significantly smaller cross-sectional area than the rest of the sample might contribute significantly more to the electrical resistance or resistivity of the material, as measured across the entire sample. While in this study we did not seek to precisely quantify the extent of this effect, we do seek to elucidate the extent of necking behavior in the samples as a function of strain, which is expected to occur in polymeric samples like these [45,46].

**Figure 7:**
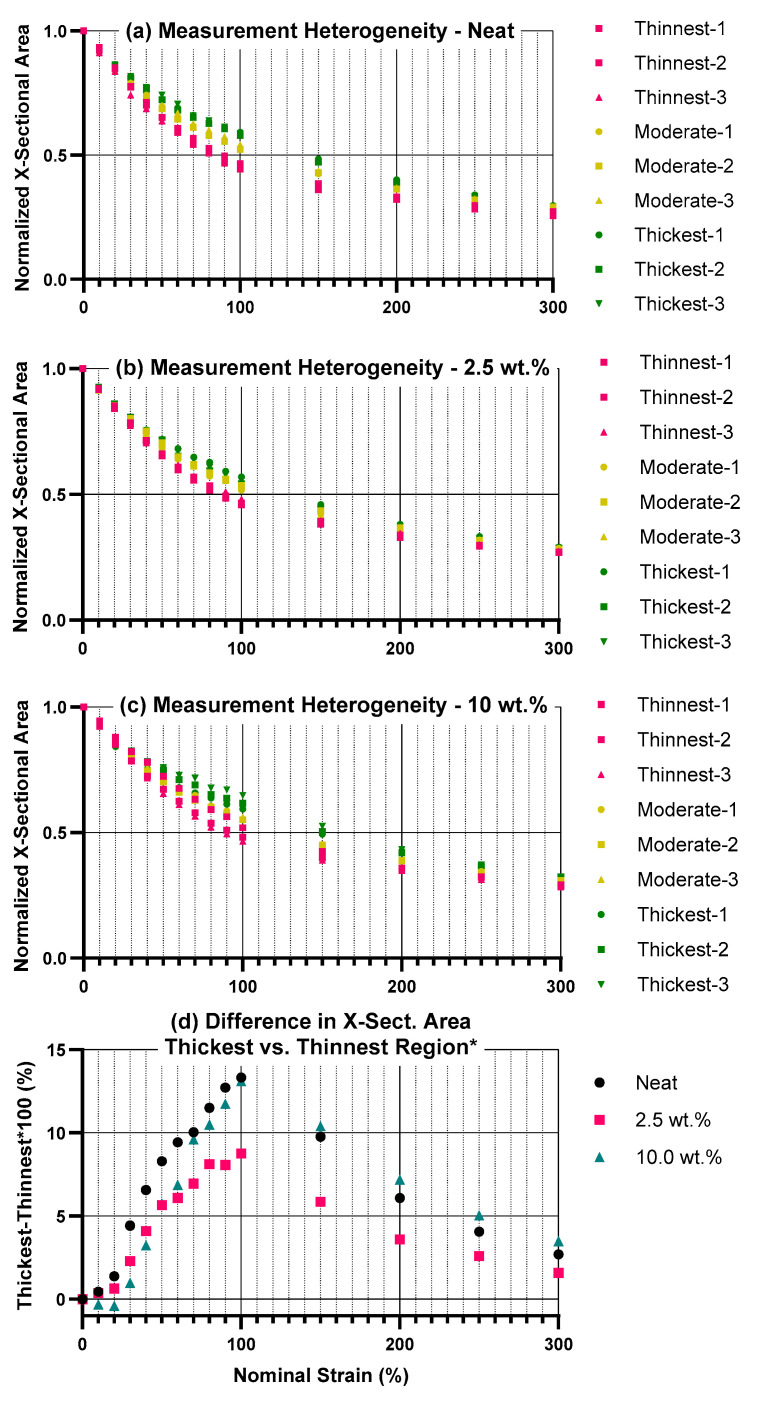
Geometrical differences across the three measurement locations, indicating necking. (**a**–**c**) Cross-sectional area is shown, normalized to initial cross-sectional area, for each sample. Red, green, and yellow data points indicate the results from the thinnest region, thickest, and moderate regions, respectively. The “thinnest” section was whichever measurement location (bottom/middle/top) had the lowest average cross-sectional area across all strains; the same logic applied for the “moderate” and “thickest” sections. (**d**) Difference of thickest and thinnest regions, with this difference represented as a percent of original cross-sectional area. * Note that since “thinnest” and “thickest” are determined based on average behavior over all strains, it is possible to have a negative difference at particular strains, as seen with the 10 wt.% results, if the region of greater initial measured thinning did not become the region dominated by necking.

Figure 7a–c show the normalized cross-sectional areas of the samples, as determined via thickness and width at the three different measurement locations for each strain setpoint (shown in Figure 1). The results from the three measurement locations are plotted separately: the thinnest region (greatest necking), the thickest region (least necking), and the third (moderate) region, as determined by the average normalized cross-sectional area over all strains. In most of the 15 total samples tested, necking occurred in one localized region—either at the top or at the bottom. In several cases, necking occurred either in the center of the sample, or at both the top and the bottom of the sample. The position of the yellow line in these results relative to the others can provide an indication of these behaviors. Figure 7d shows the difference between the thickest and thinnest regions’ cross-sectional areas, represented as a percent of original cross-sectional area. An interesting observation from these results is that there are several small negative numbers at low strain for the 10 wt.% sample, which indicates that the location of initial heterogeneity is not always where necking ultimately occurs. Thus, these negative numbers suggest we are not observing plastic instability in the traditional sense, but do indicate some slight, stable geometrical heterogeneities at low strain in the 10 wt.% sample.

These results show that no notable necking occurs in the first 10% nominal strain (<1% difference), minimal necking occurs from 10–20% nominal strain (<2% difference); above this, necking behavior increases toward a maximum around 100% strain. By 50% strain, all three material treatments displayed in Figure 7 show >5% difference in cross-sectional area between the thickest and the thinnest region. Following this maximum, the geometric differences decrease again, consistent with neck propagation behavior expected to occur in polymers [45,46]. Ultimately, these results will need to be interpreted in context of future testing applications of these materials. Nonetheless, they demonstrate that the heterogeneity in necking behavior exhibited in these polymer composites should be considered when evaluating their electrical properties as a function of large plastic strains.

### 3.4. Limitations

This study sought to quantify changes in transverse strain as a function of longitudinal strain in carbon black-UHMWPE composites, toward an understanding of how these materials’ electrical resistivity can be assessed as a function of strain. As a result, rigorous investigations of some of the effects discussed, such as necking or volumetric opening, were outside of the scope of this study. Future work could employ microCT or other imaging modalities to understand the precise change in volume of these materials due to volumetric opening. Furthermore, this study did not seek to rigorously interpret the results in the context of various plasticity theories as other authors have done [41], and instead focused on characterizing the material to the extent necessary for practical implementation of electrical resistivity measurements in long rectangular samples.

Furthermore, an intrinsic limitation of this study is that it relies on an operator’s consistent measurements. While the methods described in this study are believed to provide highly accurate measurements, as indicated by the consistent results, individual measurements are expected to vary slightly due to several factors. For the thickness measurements, a small amount of dust or other contaminants can occasionally get on the samples, or between the micrometer’s anvil and spindle, changing the measurements by up to several micrometers. For both thickness and width measurements, the precise location of the measurement could vary by up to several millimeters and influence the measurements, especially after the onset of geometric heterogeneity (necking). Finally, as viscoelastic materials, the time between the strain event and measurements could matter slightly, especially during neck propagation periods. While this effect was controlled by having a defined period of time in which measurements were taken, measurements taken later in that period tended to be smaller than measurements taken earlier. That said, these differences were minimal. While these effects are small in magnitude and not expected to significantly affect the results of this study, their presence is important to be aware of in future work to achieve precise determination of sample geometry.

## 4. Conclusions

This study investigated three primary questions, all related to the geometric deformation of electrically conductive composites of UHMWPE under large plastic strains. Thus, here we have investigated (1) the ability for volume conservation to be used to predict transverse sample geometries in strained samples based on initial geometry and longitudinal strain, (2) the influence of filler concentration on sample geometry under large plastic deformations, and (3) the necking behavior of these composite polymer materials. Regarding (1), it was observed that the materials’ thicknesses and widths remained within 2% of those predicted by volume conservation up to 30% nominal strain in both the composites and the neat control, and within 5% up to 100% nominal strain. Materials with higher filler loading, e.g., 10 wt.%, experienced an increase in deviation from the predicted line, particularly at higher strains. These results can thus inform how well volume conservation principles can predict sample cross-sectional areas in strained conditions during future work to assess the intrinsic electrical properties of these materials. Along similar lines, for (2), it was observed that at higher filler concentrations and higher strains, measured geometries tended to be larger in the composites as compared to the neat control material. This was demonstrated by both the thickness and width data and the calculated volume data, demonstrating increases in the magnitude of these geometric parameters relative to neat controls with increasing carbon concentration and strain. This work provided evidence in favor of the hypothesis that this difference would be due in large part to volumetric opening in the composite materials with high filler loading. Finally, regarding (3), in this study, we observed necking behavior presenting in a significant manner above 20% nominal strain, followed by neck propagation as expected in this type of material. The results of this study thus lay the foundation for further characterization of these electrically-conductive composites, and to determine their intrinsic electrical properties as a function of strain in particular.

## Figures and Tables

**Table 1:** Percent difference between experimental thickness (t) and width (w) as compared to thickness and width as predicted by volume conservation principles applied to initial sample geometry. All computations were performed using the average of the three experimental data points.

	Neat		2.5 wt.%		5 wt.%		7.5 wt.%		10 wt.%	
Nom. Strain	t	w	t	w	t	w	t	w	t	w
0%	0.0%	0.0%	0.0%	0.0%	0.0%	0.0%	0.0%	0.0%	0.0%	0.0%
10%	1.0%	0.8%	0.0%	0.8%	−0.1%	0.7%	0.3%	0.3%	−0.9%	1.2%
20%	1.1%	1.1%	0.5%	1.1%	0.2%	1.1%	0.9%	0.8%	0.0%	1.2%
30%	1.1%	1.2%	1.2%	1.4%	0.5%	1.4%	1.0%	1.1%	0.5%	1.5%
40%	1.5%	1.4%	1.0%	1.5%	0.4%	1.5%	1.5%	1.5%	1.2%	2.2%
50%	1.6%	1.8%	1.1%	1.7%	0.8%	1.6%	2.2%	1.9%	1.7%	2.6%
60%	2.0%	1.7%	0.3%	1.9%	0.9%	2.1%	2.7%	2.2%	2.0%	2.8%
70%	1.9%	1.7%	0.5%	2.1%	1.1%	2.3%	2.4%	2.3%	3.0%	3.3%
80%	2.4%	1.9%	0.0%	2.7%	1.3%	2.4%	2.4%	2.8%	2.4%	3.7%
90%	2.3%	2.2%	0.3%	2.5%	1.2%	2.8%	2.7%	3.2%	3.0%	4.6%
100%	2.1%	2.2%	0.6%	2.3%	1.5%	2.8%	3.1%	3.5%	3.5%	4.5%
150%	3.4%	3.2%	1.6%	3.4%	2.2%	4.0%	4.0%	5.1%	4.5%	6.0%
200%	4.3%	3.9%	2.6%	4.1%	3.1%	5.0%	5.2%	6.5%	5.7%	7.6%
250%	5.1%	4.9%	3.4%	5.2%	3.9%	6.2%	6.1%	8.2%	6.9%	9.4%
300%	6.1%	6.0%	4.6%	6.6%	5.1%	7.7%	7.3%	9.2%	8.5%	9.7%

## Data Availability

The original contributions presented in this study are included in the article. Further inquiries can be directed to the corresponding author.

## Supplementary Materials

The following supporting information can be downloaded at: https://www.mdpi.com/article/10.3390/ma18112542/s1, One item is included in the Supplementary Materials: Table S1: Sample-to-sample standard deviation (SD) and coefficient of variation for all experimental thickness and width measurements.
