# Supplementary material for: Tensile Deformation and Transverse Strain Behavior of Carbon Black-UHMWPE Composites
Source: Materials (Basel). 2025 May 28;18(11):2542. doi: 10.3390/ma18112542 (PMC12155802; doi:10.3390/ma18112542)
Supplement: Supplementary file 1 [file materials-18-02542-s001.zip › materials-3631177-supplementary.pdf]

Supplementary Material

Table S1: Sample-to-sample standard deviation (SD) and coefficient of variation for all experimental thickness and width measurements. Standard deviation was computed in Microsoft Excel using the sample standard deviation function, STDEV.S(). Coefficient of variation at a particular value of strain takes the SD, divides it by the mean thickness or width value at that strain, and multiplies it by 100. These results correspond to data displayed in the main text – Figure 2, in particular.

Nominal Strain (%)	**Sample-to-Sample Standard Deviation of Thickness (n=3 samples)**	**Coefficient of Variation of Thickness (SD as a % of mean thickness)**	**Sample-to-Sample Standard Deviation of Width (n=3 samples)**	**Coefficient of Variation of Width (SD as a % of mean width)**
Neat				
	Thickness		***Width***	
0	0.0006	0.29	0.013	0.41
10	0.0002	0.10	0.015	0.48
20	0.0002	0.10	0.016	0.52
30	0.0008	0.47	0.020	0.69
40	0.0008	0.45	0.019	0.66
50	0.0002	0.12	0.029	1.06
60	0.0004	0.24	0.036	1.35
70	0.0007	0.44	0.029	1.14
80	0.0003	0.22	0.034	1.36
90	0.0007	0.46	0.036	1.46
100	0.0010	0.66	0.042	1.75
150	0.0009	0.67	0.038	1.79
200	0.0013	1.10	0.036	1.81
250	0.0010	0.90	0.047	2.55
300	0.0012	1.08	0.040	2.32
2.5 wt.%				
	Thickness		***Width***	
0	0.0025	1.26	0.008	0.26
10	0.0023	1.22	0.007	0.22
20	0.0030	1.64	0.008	0.28
30	0.0029	1.63	0.008	0.29
40	0.0030	1.74	0.013	0.48
50	0.0031	1.86	0.014	0.51
60	0.0026	1.63	0.018	0.69
70	0.0031	2.00	0.019	0.74
80	0.0020	1.35	0.007	0.28
90	0.0030	2.04	0.022	0.91
100	0.0030	2.11	0.035	1.46
150	0.0032	2.46	0.033	1.52

200	0.0030	2.51	0.037	1.85
250	0.0038	3.45	0.032	1.75
300	0.0034	3.18	0.034	1.95
5 wt.%				
	Thickness		***Width***	
0	0.0005	0.25	0.002	0.06
10	0.0008	0.44	0.007	0.22
20	0.0007	0.38	0.007	0.23
30	0.0011	0.60	0.007	0.23
40	0.0013	0.79	0.010	0.34
50	0.0003	0.20	0.013	0.49
60	0.0003	0.21	0.022	0.82
70	0.0002	0.12	0.007	0.27
80	0.0003	0.22	0.012	0.47
90	0.0004	0.26	0.021	0.88
100	0.0005	0.35	0.019	0.78
150	0.0011	0.82	0.028	1.29
200	0.0010	0.85	0.024	1.21
250	0.0008	0.75	0.026	1.39
300	0.0012	1.11	0.026	1.49
7.5 wt.%				
	Thickness		***Width***	
0	0.0012	0.58	0.007	0.20
10	0.0015	0.78	0.003	0.11
20	0.0015	0.81	0.006	0.19
30	0.0010	0.56	0.010	0.35
40	0.0007	0.39	0.011	0.38
50	0.0020	1.20	0.013	0.49
60	0.0016	0.96	0.023	0.85
70	0.0016	1.04	0.012	0.47
80	0.0013	0.88	0.019	0.74
90	0.0013	0.89	0.020	0.82
100	0.0015	1.02	0.024	0.98
150	0.0018	1.38	0.025	1.15
200	0.0018	1.50	0.032	1.57
250	0.0022	1.91	0.021	1.11
300	0.0022	2.02	0.020	1.11
10 wt.%				
	Thickness		***Width***	
0	0.0004	0.20	0.005	0.16
10	0.0006	0.30	0.015	0.47
20	0.0004	0.21	0.002	0.06
30	0.0002	0.11	0.010	0.35

40	0.0005	0.30	0.012	0.42
50	0.0010	0.58	0.004	0.14
60	0.0015	0.94	0.010	0.38
70	0.0017	1.05	0.004	0.15
80	0.0015	0.99	0.010	0.38
90	0.0017	1.12	0.008	0.31
100	0.0013	0.91	0.011	0.44
150	0.0020	1.51	0.017	0.78
200	0.0014	1.13	0.014	0.68
250	0.0020	1.76	0.020	1.04
300	0.0013	1.15	0.025	1.38
